# A Simple and Safe Method for Checking the Position of Central Venous Catheters—A New and Reliable Threshold for Right Atrial Swirl Sign in Microbubbles Tests

**DOI:** 10.3390/jcm13061657

**Published:** 2024-03-14

**Authors:** Lukas Ley, Hossein Ardeschir Ghofrani, Pascal Klingenberger, Tilo Niemann, Jens Allendörfer, Dirk Bandorski

**Affiliations:** 1 Campus Kerckhoff, Justus-Liebig-University Giessen, 61231 Bad Nauheim, Germany; lukas.m.ley@med.uni-giessen.de; 2 Department of Radiology, Kantonsspital Baden, 5404 Baden, Switzerland; tilo.niemann@ksb.ch; 3 Universities of Giessen and Marburg Lung Center (UGMLC), 35392 Giessen, Germany; ardeschir.ghofrani@innere.med.uni-giessen.de; 4 Neurologische Klinik Bad Salzhausen, ASKLEPIOS, 63667 Nidda, Germany; p.klingenberger@asklepios.com (P.K.); j.allendoerfer@asklepios.com (J.A.); 5 Faculty of Medicine, Semmelweis University Campus Hamburg, 20099 Hamburg, Germany

**Keywords:** central venous catheter position, right atrial swirl sign, microbubbles test, push-to-bubbles time

## Abstract

**Background**: Central venous catheters (CVCs) are indispensable tools in intensive care and emergency medicine. CVC malpositions still occur frequently and can cause various complications leading to increased patient mortality. A microbubbles test (MBT) can be used to confirm correct CVC positioning. However, there is serious doubt regarding whether the currently applied threshold of a 2 s push-to-bubbles time (PTB time) for rapid atrial swirl sign (RASS) in an MBT is reliable and accurate. The aim of the present study was to prove the quality of a new threshold: 1 s. **Methods**: Consecutive patients who were admitted to the intensive care unit (ICU) in a German neurological specialist hospital from 1 March 2021 to 20 July 2022 were enrolled. After ultrasound-guided CVC insertion, an MBT was performed, PTB time was measured, and RASS was interpreted. Additionally, a chest X-ray (CXR) was requested to check CVC position. **Results**: A total of 102 CVCs (98% jugular and 2% subclavian) were inserted in 102 patients (38% female and 62% male; median age: 66 years). Negative RASS (PTB time > 1 s) was observed in 2 out of 102 patients, resulting in an echocardiographic malposition rate of 2.0%. CXR confirmed the echocardiographic results. After correcting CVC position in the initially malpositioned CVCs, the PTB time was <1 s (positive RASS). The MBT protocol took about 0.5 min on average, while the CXR results were all available within 30 min. Sensitivity, specificity, and positive and negative predictive value were each 100% for the detection of CVC malpositions via an MBT using a threshold of 1 s compared to CXR. **Conclusions**: A new threshold of a 1 s PTB time for RASS in an MBT could detect CVC malpositions with excellent quality compared to CXR. Since the MBT was fast and safe and could be performed at the bedside, we propose that an MBT with the new and reliable threshold of 1 s should be routinely used in patient care.

## 1. Introduction

Since its introduction by Seldinger in 1953, the central venous catheter (CVC) has become an indispensable tool in intensive care and emergency medicine for various indications [1]. In intensive care units (ICUs), an estimated 90% of patients receive a CVC [2]. As ultrasound-guided CVC insertion increases quality and safety compared to the anatomical landmark technique, its use is recommended by the American Society of Anaesthesiologists (ASA) [3,4,5]. Despite the ubiquitous availability of ultrasound technology, CVC misplacement still occurs in up to 17.6% of patients [6]. In up to 12.3%, malposition-related complications such as arrhythmias, damage to cardiac structures, pericardial tamponade, thrombosis with consecutive infection, and pneumothorax can occur [7,8,9,10]. These complications can lead to increased patient morbidity and mortality, as well as the placement of an economic burden on the health care system [11]. Although a chest X-ray (CXR) is not capable of adequately confirming correct CVC positioning, it is still partially recommended by the ASA and is carried out in most hospitals, serving as a gold standard [5,12,13]. A microbubbles test (MBT) is another modality, and it has proven to be able to verify correct CVC positioning [2,14,15,16,17,18,19,20,21,22,23,24]. In an MBT, after the rapid injection of a saline air mixture into the CVC, the result of this manoeuvre is observed in transthoracic echocardiography (TTE) images. If the catheter is positioned correctly, numerous bubbles with linear flow within 2 s coming from the superior vena cava (SVC) can be detected (positive rapid atrial swirl sign (RASS)). The absence of bubbles, the appearance of only a few bubbles, the appearance of bubbles after >2 s, or numerous bubbles with turbulent flow in the right atrium (RA) constitute what is called negative RASS [21,22]. However, some studies suggest that the currently applied threshold of 2 s until the appearance of microbubbles is too high and inaccurate [16,18,19,20,21]. The aim of the present study was to prove the quality of a new threshold for an MBT based on our clinical experience. We hypothesised that a threshold of 1 s may be more reliable.

## 2. Material and Methods

### 2.1. Study Design

The present study was conducted as a unicentre, prospective study in an ICU of a German neurological specialist hospital. Consecutive patients who were admitted to the ICU from 1 March 2021 to 20 July 2022 were reviewed regarding the exclusion criteria (emergency insertion of a CVC, absolute contraindications for CVC insertion, and denial consent for the study). All patients without exclusion criteria were included. This study was approved by the Ethics Committee of the Department of Medicine at Justus-Liebig-University (reference number: 250/20, dated 21 October 2020) and was registered at ClinicalTrials.gov (ID: NCT04630236, Protocol ID: CVC061120).

### 2.2. Primary and Secondary Outcome Parameters

The primary outcome parameter was the effectiveness of a threshold of <1 s for PTB time in MBT indicated by sensitivity, specificity, positive predictive value (PPV), and negative predictive value (NPV) in comparison to CXR. Secondary outcome parameters were the time required for the procedure and the rate of technical success, especially with respect to obtaining sufficient echocardiographic views.

### 2.3. Central Venous Catheter Insertion 

The internal jugular vein (IJV) or the subclavian vein (SV) was cannulated with a three- or four-lumen CVC (Arrowg+ard Blue Plus^®^, 7 or 8.5 F × 20 cm, Teleflex Inc., Wayne, PA, USA) using the Seldinger technique [1]. All punctures were performed with the patient in the supine position under sterile ultrasound control (Vivid S6, GE HealthCare, Chicago, IL, USA).

### 2.4. Microbubbles Test

The procedure of the MBT has been described before [22]. After checking the continuity of the CVC lumen, the first interventionalist visualised the right side of the heart using TTE (Vivid S6, GE HealthCare, Chicago, IL, USA). The subxiphoid view was considered the first choice and the apical four-chamber view was the second choice for visualising the RA. Meanwhile, the second interventionalist prepared a 10 mL syringe filled with 9 mL of 0.9% saline and 1 mL of air. Microbubbles were created by shaking the syringe vigorously or by mixing two separate injections via a three-way tap, exchanging the saline air mixture between syringes. After removing all visible air, the mixture was then rapidly injected into the distal lumen of the CVC, and the reaction in the RA was observed via TTE. The MBT was interpreted according to Table 1, and the time from injection to visibility of bubbles in the RA (push to bubbles time, PTB time) was measured and documented in intervals of whole seconds (<1 s, <2 s, etc.). RA position of the CVC was excluded based on TTE.

### 2.5. Chest X-ray

**Figure 1:**
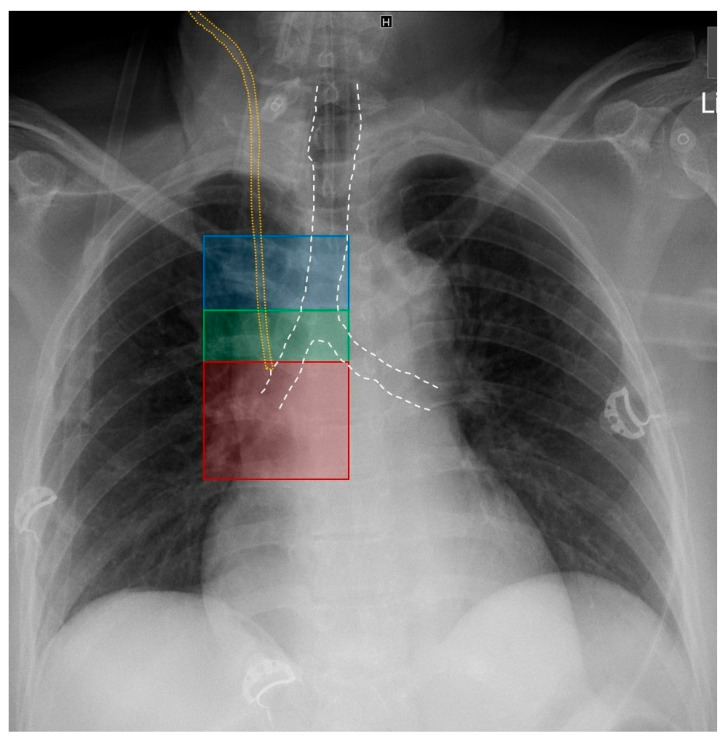
Radiological zones of CVC positions. Annotations: blue = zone 1: above carina level (superior vena cava), green = zone 2: at carina level (terminal superior vena cava), red = zone 3: below carina level (superior vena cava/right atrium transition), white: trachea contour, and yellow: CVC contour.

Immediately after the CVC was inserted, a CXR (OPTIMA XR 240 AMX, GE HealthCare, Chicago, IL, USA) was requested to check CVC position and exclude a pneumothorax. CXR was assessed by an experienced radiologist and intensive care physician. The radiological CVC position was assigned to one of three zones, which were modified according to Fletcher and Bodenham [25] and interpreted according to Table 1 and Figure 1.

### 2.6. Statistics

A statistical analysis was performed using SPSS Statistics (Version 27, IBM, Armonk, NY, USA). Nominal variables are presented as numbers and percentages. Since the Shapiro–Wilk test revealed that the variables were non-normally distributed continuous variables, they are presented as medians and interquartile ranges (IQRs). We calculated sensitivity, specificity, PPV, and NPV using a four-fold table. Sensitivity, specificity, PPV, and NPV were calculated according to the following assumptions: A CVC malposition detected via MBT and CXR was considered a true-positive event, and a correct CVC position detected via MBT and CXR was considered a true-negative event. A CVC malposition misidentified as a correct CVC position only via MBT (wherein CXR detected an actual malposition) was considered a false-negative event, and a correct CVC position misidentified as malpositioned CVC only via MBT (wherein CXR detected an actual position) was considered a false-positive event.

## 3. Results

A total of 102 patients (38% female), with a median age of 66 years, were included in this study (Table 2). A total of 102 CVCs were inserted, all of which were ultrasound-guided. No patients had to be excluded due to inadequate echocardiographic image quality (the secondary outcome parameter). All MBTs were carried out in the first attempt. Out of 102 MBTs, negative RASS (PTB time > 1 s) was observed in two cases, resulting in an echocardiographic malposition rate of 2.0%. In both malpositions, the occurrence of bubbles was delayed (PTB time > 2 s). Both CVC misplacements were identified as kinked catheters via a CXR, with one in the left and one in the right SV. In all the correctly positioned CVCs, PTB time was consistently <1 s (positive RASS). Moreover, after the position of the initially malpositioned CVCs was corrected, PTB time was <1 s as well. CXR confirmed the echocardiographic results and detected two malpositions in the same patients (corresponding to a 2.0% radiological malposition rate). The radiological distribution of correctly placed CVC tips can be seen in Table 2. No pneumothorax was detected via a CXR. The MBT protocol took about 0.5 min on average (the secondary outcome parameter), while the CXR results were all available within 30 min. We calculated the sensitivity, specificity, PPV, and NPV of each 100% accurate score (constituting the primary outcome parameters) for the detection of CVC malpositions via an MBT using a threshold of a 1 s PTB time compared to CXR. No complications were observed in any patient.

## 4. Discussion 

### 4.1. Common Modalities for Confirmation of Central Venous Catheter Position and Exclusion of Pneumothorax

Catheter malpositioning occurs in up to 17.6% of all patients receiving a CVC, and pneumothoraxes occur in 0.6–1.5% [6,7,26]. To prevent complications due to malpositions, the correct CVC position should be confirmed, and a pneumothorax should be ruled out after CVC insertion. For this purpose, CXR, electrocardiography (ECG), lung ultrasound (US), and TTE are the modalities most commonly used. Although CXRs continue to be partially recommended by the ASA and are considered the gold standard in most hospitals, a CXR cannot adequately confirm the correct CVC position nor adequately exclude pneumothorax [5,12,13,27,28,29]. The periprocedural ECG method for the confirmation of the catheter tip position is based on the analysis of P-wave morphology. It is not able to distinguish between an intra-arterial, intravenous, and extravascular CVC location. There is no consensus on the interpretation of P-wave morphology and its anatomical correlate, and this technique cannot be used in the absence of P waves (e.g., in cases of atrial fibrillation or cardiac pacing) [30,31,32,33,34,35,36]. The most accurate method for confirming CVC position is transoesophageal echocardiography (TEE) [37,38]. However, its invasiveness makes it unsuitable for everyday use. Overall, the combination of TTE and lung US (both standard procedures in ICUs) appears to be the most promising modality for the rapid and effective detection of CVC malpositions and iatrogenic pneumothoraxes [2,39,40]. Lung US can detect pneumothoraxes with a sensitivity and specificity of 100%, is more reliable than a CXR, and offers (almost) the same quality as a computed tomogram (CT) for this purpose [6,41,42,43,44]. Many studies demonstrated the practicality of TTE for the confirmation of CVC position. On the one hand, TTE can be useful for confirming the correct guide wire position before catheter insertion [45,46,47]. However, it should be considered that changes in guide wire position may occur while inserting a catheter into its final position. Moreover, the guide wire can be confused with various structures in some US views (the aorta, the inferior vena cava wall, pacemaker wires, or other catheters) [48]. On the other hand, further studies have shown that injection of common saline under TTE monitoring can rapidly determine the correct CVC position [49,50]. However, there are constellations where this non-contrasted method reaches its limit. Sometimes, only the use of an MBT can detect every catheter malposition [19,22].

### 4.2. The Current Study Situation on Push-to-Bubbles Time in Microbubbles Tests

The effectiveness of an MBT has been investigated in numerous studies (Table S1) [2,14,15,16,17,18,19,20,21,22,23,24]. However, sensitivity and specificity for the detection of CVC malpositions using an MBT range from 22 to 100% and 90 to 100% (PPV: 66–100%, NPV: 70–100%) [15,23]. On the one hand, this could be due to the choice of TTE views. Corradi et al. reported poor sensitivity, specificity, PPV, and NPV (22%, 90%, 66%, and 70%) for the apical four-chamber view and significantly better sensitivity, specificity, PPV, and NPV (97%, 90%, 83%, and 98%) for the subxiphoid view [15]. On the other hand, this could be due to a potentially inaccurate current threshold for PTB time. For example, Cortellaro et al. found poor sensitivity for the detection of CVC malpositions using an MBT (33%), which was caused by four false negatives (misplaced CVCs considered correctly positioned) in a total of 71 patients. In these four false negatives, the CVCs were all inserted in the jugular, and the PTB time was <2 s. In CXRs, the CVCs were found in the surrounding subclavian and brachiocephalic veins or the RA [16]. In other studies that performed a complete MBT, the sensitivity and specificity for the detection of CVC malpositions were 75–100% and 94–100% (PPV: 70–100%, NPV: 99–100%) [2,17,19,21,22,23,24]. This moderate sensitivity and the moderate positive predictive value were also significantly influenced by false negatives (misplaced CVCs considered correctly positioned). False negatives were commonly found in the surrounding veins (left brachiocephalic vein and right internal jugular vein) [21,24]. Malpositions in the surrounding veins could have been found by using a lower threshold (e.g., 1 s) because the PTB time of their false negatives may have been in the range of 1–2 s. RA malpositions could have been detected through the sonographic visualization of the catheter tip [39]. Evidently, with its current threshold, the MBT seems incapable of identifying all incorrect CVC positions in every study. In fact, some studies already suggested that the currently applied threshold of 2 s is too high and inaccurate [16,18,19,20,21]. Cortellaro et al. reported four false negatives (misplaced CVCs considered correctly positioned), all of which had a PTB time < 2 s [16]. Gidaro et al. conducted a study including 132 patients and investigated the quality of the MBT in sensing CVC malpositions compared to CXRs while inserting peripheral venous catheters. Even though the catheters were not in a completely central position, 29.5% of the catheters had a PTB time ≤ 2 s [18]. Iacobone et al. performed an MBT on 42 patients receiving centrally and peripherally inserted central catheters (CICC and PICC). They specified an average PTB time of 0.89 ± 0.33 s for their true-negative (correctly positioned catheters considered correctly positioned) CICCs, which indicates that 84.1% of the correctly positioned CICCs had a PTB time < 1.22 s [19]. Meggiolaro et al. conducted a similar study on 105 patients receiving CVCs. They found an average PTB time of 0.2 s for correctly positioned CVCs and an average PTB time of 1.4 s for incorrectly positioned CVCs. In their study only 29% of the malpositioned CVCs had a PTB time of >2 s. A threshold of 0.5 s had a sensitivity of 100% and a specificity of 99% for CVC malpositions [20]. Lastly, Weekes et al. reported a mean PTB time of 1.1 s [21]. All these studies support our decision to evaluate a lower threshold for RASS in an MBT. In the present study, it was shown that microbubbles appeared after >2 s in all malpositioned CVCs, but in all correctly positioned CVCs, bubbles already appeared after <1 s. For a threshold of 1 s, we calculated a sensitivity, specificity, PPV, and NPV of 100%. However, there are also studies that report excellent sensitivity, specificity, PPV, and NPV (each being 100%) when using a threshold of 2 s [17,19,23]. Wen et al. and Duran-Gehring et al. did not indicate a PTB time in their studies [17,23]. Thus, all their true negatives (correctly positioned CVCs considered correctly positioned) could have been true negatives even with a threshold of 1 s. Iacobone et al. reported an average PTB time of 0.89 ± 0.33 s for their CICCs recognized as true negatives. Based on the mean and standard deviation, an estimated 70% of their true negatives could have had a PTB time < 1 s, and 84.1% could have had a PTB time < 1.22 s [19]. This leads to several unanswered questions: Is a threshold of 1 s really optimal? Does the optimal threshold lie between 1 and 2 s? How accurate can PTB time be measured in real life without specific devices? These should be clarified in further, preferably multicentre and more extensive studies. However, since our threshold has excellent diagnostic accuracy and as we believe it is better to use a safer and stricter threshold until final results are available, we recommend the use of a 1 s PTB time threshold for RASS in an MBT in order to avoid false negatives (missed CVC malpositions).

### 4.3. Clinical Implementation of Micro Bubbles Test

Microbubbles tests are inexpensive, non-invasive, easy to learn, largely examiner-independent, seem to be safe, and can be performed at the bedside [2,19,23,51]. The current study situation suggests that examinations to exclude CVC malpositions beyond an MBT should only be performed if discrepancies in an MBT (e.g., negative RASS) occur or the quality of echocardiographic views is inadequate [2,20]. The possible further procedure depends on the suspected location of the misplaced catheter. There are many potential ways to distinguish between arterial and venous catheter malpositions, which are common in clinical practice. By inspecting blood colour, blood pulsatility, arterial blood gases, pressure curves, and pressure measurements, arterial and venous CVC positions can be distinguished [32]. Ideally, arterial misplacements can be avoided by using ultrasound-guided CVC insertion [3,4]. When safe, an arterially placed catheter must be (surgically) removed [52,53]. For a venous malposition, a vascular ultrasound of the neighbouring vessels could be one of the first modalities to perform to search for a misplacement in the surrounding veins [22]. When a misplaced catheter is found, an interventionalist can decide between removing or adjusting the CVC. If lung US is not applicable, CT or CXR could then be performed to exclude pneumothorax.

### 4.4. Limitations

Despite appearing beneficial, for up to 10.8% of patients, an MBT cannot be performed [22]. In most cases, this is because of poor echocardiographic image quality, mostly for patients with a high body mass index (BMI) [2,14]. However, we were able to acquire sufficiently good echocardiographic images of all the patients (a 100% technical success rate). Moreover, there are some factors (e.g., acute heart failure, long catheter length, a large catheter diameter, slow injection speed, and inaccuracy in the estimation/measurement of PTB time) that may prolong PTB time despite correct catheter positioning [15,21]. Another problem arises with the absence of microbubbles. The absence of microbubbles could indicate both an arterial misplacement or a venous misplacement distant from the right side of the heart [21]. In this case, the procedure mentioned above could help. Our study has some limitations. One limitation is the lack of a comparison group tested using the currently applied threshold of <2 s. We decided to use the already-available comprehensive data on the old threshold of <2 s and thus extensively compare our results (with a <1 s threshold) with those of previous studies (Table S1). Moreover, the results’ transferability to the general population is limited by the low number of patients we included (*n* = 102), the unbalanced insertion site of the CVCs (98% jugular and 2% subclavian), the study design (single-centre, with a potential selection bias), and the exclusive inclusion of adults (offering questionable transferability to paediatric patients). In addition, our data on PTB time were based on measurements taken at intervals of whole seconds (<1 s, <2 s, etc.). However, we deliberately decided not to measure PTB time exactly, as there are currently no suitable, ubiquitously available measuring devices for this purpose, and thus we wanted to imitate the real-life situation in the ICU. Furthermore, we compared only the estimated time required for our MBT protocol to that of a CXR. However, the potential time reduction of the MBT protocol has been proven in many studies before [2,14,16,17,20,22,23,24]. We also did not perform lung US for pneumothorax exclusion. This was due to the fact that we wanted to focus on testing the new threshold of 1 s for RASS in this study, but the superiority of lung US has also been proven before [41,42,43]. Another limitation of the present study is the chosen comparative modality of a CXR, which is not fully appropriate [12,13,27,28,29]. Although using TEE as a comparative modality would be more appropriate, it is too invasive to be applied to a regular study cohort [37,38]. Furthermore, most of the studies conducted on MBTs so far chose a CXR as a comparative modality [2,14,16,17,18,19,20,21,22,23,24].

## 5. Conclusions

The present study demonstrated that a threshold of a 1 s PTB time for RASS in an MBT was able to detect CVC malpositions with excellent sensitivity, specificity, PPV, and NPV (each 100%) compared to a CXR. In addition, the MBT was fast (0.5 min) and safe and could be performed at the bedside for all patients in the first attempt. Therefore, we propose that MBTs with this new and reliable threshold of 1 s should be routinely used in patient care. Further, more extensive and multicentre studies are required to underpin this new threshold.

## Figures and Tables

**Table 1:** Interpretation of microbubbles test.

RASS	Echocardiographic Correlate	Anatomical Correlate	Radiological Correlate	CVC Position
Positive RASS	Rapid (PTB time < 1 s) appearance of multiple bubbles in the RA	CVC in SVC or RA	Zone 1: above carina level (SVC) Zone 2: at carina level (terminal SVC) Zone 3: below carina level (SVC/RA transition)	Correct position
Negative RASS	Slow (PTB time > 1 s) appearance and/or appearance of few bubbles in the RA	CVC in venous system (veins other than SVC or RA)	Out of zones 1–3	Malposition
	No appearance of bubbles in the RA	CVC in arterial system or venous system distant from heart	Out of zones 1–3	Malposition

Annotations: CVC: central venous catheter, PTB: push to bubbles, RA: right atrium, RASS: rapid atrial swirl sign, and SVC: superior vena cava.

**Table 2:** Characteristics of the study cohort.

Patients (n)	102
CVCs (n)	102
Age (median, interquartile range, years)	66 (57–76)
Female/male (%)	38, 62
CVC insertion site: LIJV (%)	53.9 (55/102)
CVC insertion site: RIJV (%)	44.1 (45/102)
CVC insertion site: LSV (%)	2.0 (2/102)
Ultrasound-guidance rate (%)	100
Average number of attempts	1
Setting	ICU (100%)
Mechanically ventilated (%)	100
TTE views	Subxiphoid (100%)
TTE: technical success (%)	100 (102/102)
Correctly placed catheters/RASS positive (TTE, %)	98.0 (100/102)
Misplaced catheters/RASS negative (TTE, %)	2.0 (2/102)
Correctly placed catheters (CXR, %)	98.0 (100/102)
Correctly placed catheters in zone 1 (CXR, %)	30.4 (31/102)
Correctly placed catheters in zone 2 (CXR, %)	26.5 (27/102)
Correctly placed catheters in zone 3 (CXR, %)	43.1 (44/102)
Misplaced catheters (CXR, %)	2.0 (2/102)
Pneumothorax rate (CXR, %)	0 (0/102)
Time to TTE result (min)	About 0.5
Time to CXR results (min)	Within 30
PTB time when RASS was positive (s)	<1
PTB time when RASS was negative (s)	>2
Sensitivity (%)	100
Specificity (%)	100
PPV (%)	100
NPV (%)	100

Annotations: CVC: central venous catheter, CXR: chest X-ray, ICU: intensive care unit, LIJV: left internal jugular vein, LSV: left subclavian vein, NPV: negative predictive value, PPV: positive predictive value, PTB: push to bubbles, RASS: rapid atrial swirl sign, RIJV: right internal jugular vein, and TTE: transthoracic echocardiography.

## Data Availability

The data presented in this study are completely contained within the article or Supplementary Materials.

## Supplementary Materials

The following supporting information can be downloaded at https://www.mdpi.com/article/10.3390/jcm13061657/s1. Table S1. The current study situation regarding microbubbles tests.
