# Supplementary material for: A Simple and Safe Method for Checking the Position of Central Venous Catheters—A New and Reliable Threshold for Right Atrial Swirl Sign in Microbubbles Tests
Source: J Clin Med. 2024 Mar 14;13(6):1657. doi: 10.3390/jcm13061657 (PMC10970920; doi:10.3390/jcm13061657)
Supplement: Supplementary file 1 [file jcm-13-01657-s001.zip › jcm-2923055-supplementary.pdf]

Table S1. The current study situation on micro bubbles test.

Author	Amir and Knio et al.	Corradi et al.	Cortellaro et al.	Duran-Gehring et al.	Gidaro et al.	Iacobone et al.
Year, country	2017, United States	2022, Italy	2014, Italy	2014, United States	2021, Italy	2020, Italy
Patients (catheters, n)	137 (137)	111 (111)	71 (71)	46 (46)	125 (125)	42 (42)
Setting	OR (80%), ICU (20%)	OR (100%)	ED (100%)	ED (100%)	-	ICU, Vascular Access Unit
Catheter insertion site (%)	RIJV: 89.7, LIJV: 5.1, RSV: 0.7, LSV: 4.4	IJV: 100	RIJV: 62, LIJV: 24, RSV: 10, LSV: 4	IJV: 91, SV: 9	Basilic vein: 63, brachial vein: 35, cephalic vein: 2	IJV: 73.8, basilic vein: 14.3, axillary vein: 7.1, brachial vein: 4.8
TTE views	Subcostal, apical	Subcostal, apical	Subcostal, apical	Subxiphoid	Subcostal, apical	Subcostal, apical
Misplaced catheters (TTE, %)	2.4 (3/124)	32 (36/111)	4.2 (3/71)	6.5 (3/46)	-	all: 4.8 (2/42), CICC: 6.3 (2/34)
Lung US views	All intercostal spaces on MCL	Not applied	Not applied	Anterior chest	Not applied	On PSL and laterally to the AAL in 3rd to 5th intercostal space
Pneumothorax rate (Lung US, %)	0 (123/123)	-	-	4.4 (2/46)	-	0
Comparative method	CXR	TEE (and CXR)	CXR	CXR	CXR	CXR
Misplaced catheters (CXR, %)	0.7 (1/137)	11 (12/111), TEE: 33 (37/111)	8.5 (6/71)	6.5 (3/46)	-	all: 4.8 (2/42), CICC: 6.3 (2/34)
Pneumothorax rate (CXR, %)	0 (0/137)	-	-	4.4 (2/46)	-	-
Push to bubbles time (s)	-	-	-	-	Overall: 2.3 ± 0.8	Positive RASS: 0.89 ± 0.33 (CICC), 1.1 ± 0.20 (PICC) Negative RASS: 3.65 ± 2.00 (CICC)
Sensitivity, specificity (%)	-	Subcostal: 97, 90; apical: 22, 94	33, 98	100, 100	-	100, 100
PPV, NPV (%)	-	Subcostal: 83, 98; apical: 66, 70	67, 94	100, 100	-	100, 100

**Annotations**	Combination of RASS and use of US to locate guidewire	-	-	-	Despite using peripheral catheters in no central position<br>29.5% had MBT ≤ 2.0 s	32 CICC, 8 PICC

**Author**	**Korsten et al.**	**Meggiolaro et al.**	**Vezzani et al.**	**Weekes et al.**	**Weekes et al.**	**Wen et al.**
**Year, country**	2018, Germany	2015, Italy	2010, Italy	2015, United States	2014, United States	2014, Germany
**Patients (catheters, n)**	100 (100)	105 (105)	99 (99)	151 (151)	142 (135, 152)	202 (219)
**Setting**	ICU, IMC	OR (100%)	ICU	ED (89%), ICU (11%)	ED, ICU	Dialysis Center
**Catheter insertion site (%)**	RIJV: 53, LIJV: 41, RSV: 2, LSV: 4	RIJV: 66, LIJV: 9, RSV: 18, LSV: 7	IJV: 23, SV: 77	RIJV: 69.5, LIJV: 21.1, RSV: 5.3, LSV: 4.0	IJV: 79, SV: 21	IJV: 100
**TTE views**	Subcostal, apical	Subcostal, apical	Subcostal	Subcostal, apical	Subcostal, apical	Subxiphoid
**Misplaced catheters (TTE, %)**	17 (17/100)	6 (7/105, performing qualitative MBT), 8.6 (9/105, performing usual TTE and vascular US)	29.3 (29/99)	2.0 (3/135)	2.2 (3/135)	0.9 (2/219)
**Lung US views**	Not applied	Longitudinal and transversal scanning of both hemithoraces	On PSL and laterally to the AAL in 3rd to 5th intercostal space	Anterior chest (on MCL at right hemithorax, on AAL at left hemithorax)	Not applied	Not applied
**Pneumothorax rate (Lung US, %)**	-	0	4 (4/99)	0	-	-
**Comparative method**	CXR	CXR	CXR	CXR	CXR	CXR
**Misplaced catheters (CXR, %)**	12 (12/100)	13 (14/105)	28.3 (28/99)	2.7 (4/135)	3 (4/135)	0.9 (2/219)
**Pneumothorax rate (CXR, %)**	1 (1/100)	0	2 (2/99)	0	1.5 (2/136)	0
**Push to bubbles time (s)**	-	Overall: 0.18 [0.13-0.32]<br>Positive RASS: 0.17 [0.12-0.23]<br>Negative RASS: 1.4 [0.7-2.2]	-	-	Overall: 1.1 (± 0.3)<br>Negative RASS: 5.0 (± 1.7)	-
**Sensitivity, specificity (%)**	100, 94	50, 100 (performing qualitative MBT); 64, 100 (performing	96, 93	75, 100	75, 100	100, 100

		usual TTE and vascular US)					
**PPV, NPV (%)**	70, 100	100 , 93 (performing qualitative MBT); 100, 95 (performing usual TTE and vascular US)	-	100, 99	100, 99	100, 100
**Annotations**	-	Elaborate protocol with electrical measuring device and scanning of image frames	Combined MBT and vascular US of IJV and SV	-	-	-

Annotations: AAL: anterior axillary line, CICC: centrally inserted central catheter, CVC: central venous catheter, CXR: Chest X-ray, ED: Emergency Department, ICU: Intensive care unit, IJV: Internal jugular vein, IMC: Intermediate care unit, LIJV: left internal jugular vein, LSV: left subclavian vein, MBT: micro bubbles time, MCL: midclavicular line, NPV: negative predictive value, OR: operating room, PICC: peripherally inserted central catheter, PPV: positive predictive value, PSL: parasternal line, RASS: rapid atrial swirl sign, RIJV: right internal jugular vein, RSV: right subclavian vein, SV: subclavian vein, TEE: transoesophageal echocardiography, TTE: transthoracic echocardiography, US: ultrasound.
